# Experimental Drillable Magnesium Phosphate Cement Is a Promising Alternative to Conventional Bone Cements

**DOI:** 10.3390/ma14081925

**Published:** 2021-04-12

**Authors:** Philipp Heilig, Phoebe Sandner, Martin Cornelius Jordan, Rafael Gregor Jakubietz, Rainer Heribert Meffert, Uwe Gbureck, Stefanie Hoelscher-Doht

**Affiliations:** 1 Department of Trauma, Hand, Plastic and Reconstructive Surgery, University Hospital of Würzburg, Oberdürrbacherstraße 6, 97080 Würzburg, Germany; p.sandner@hotmail.com (P.S.); jordan_m@ukw.de (M.C.J.); jakubietz_r@ukw.de (R.G.J.); meffert_r@ukw.de (R.H.M.); hoelscher_s@ukw.de (S.H.-D.); 2 Department for Functional Materials in Medicine and Dentistry, University of Würzburg, Pleicherwall 2, 97070 Würzburg, Germany; uwe.gbureck@fmz.uni-wuerzburg.de

**Keywords:** magnesium phosphate cement, phytic acid, inositol hexaphosphate, drillable bone cement, tibial head depression fracture, synbones, artificial bones, biomechanical evaluation, cyclic testing, load to failure testing

## Abstract

Clinically used mineral bone cements lack high strength values, absorbability and drillability. Therefore, magnesium phosphate cements have recently received increasing attention as they unify a high mechanical performance with presumed degradation in vivo. To obtain a drillable cement formulation, farringtonite (Mg_3_(PO_4_)_2_) and magnesium oxide (MgO) were modified with the setting retardant phytic acid (C_6_H_18_O_24_P_6_). In a pre-testing series, 13 different compositions of magnesium phosphate cements were analyzed concentrating on the clinical demands for application. Of these 13 composites, two cement formulations with different phytic acid content (22.5 wt% and 25 wt%) were identified to meet clinical demands. Both formulations were evaluated in terms of setting time, injectability, compressive strength, screw pullout tests and biomechanical tests in a clinically relevant fracture model. The cements were used as bone filler of a metaphyseal bone defect alone, and in combination with screws drilled through the cement. Both formulations achieved a setting time of 5 min 30 s and an injectability of 100%. Compressive strength was shown to be ~12–13 MPa and the overall displacement of the reduced fracture was <2 mm with and without screws. Maximum load until reduced fracture failure was ~2600 N for the cements only and ~3800 N for the combination with screws. Two new compositions of magnesium phosphate cements revealed high strength in clinically relevant biomechanical test set-ups and add clinically desired characteristics to its strength such as injectability and drillability.

## 1. Introduction

Mineral bone cements have a widespread use in trauma and orthopedic surgery as a filler for bone defects after fractures or tumor resections. The application of bone cements is well established in the spine, humerus and tibia and have recently been extended to the calcaneus, hip and distal radius. The rising incidence of osteoporotic fractures due to an aging western population has further increased the clinical relevance of bone cements [1]. 

It has been shown in previous studies by Hoelscher-Doht et al., 2014 [2] and Brueckner et al., 2019 [3] that the drillability of bone cement allows a complete filling of defects while providing a biomechanically superior fracture reduction. Drillability is defined as the ability of a cohesive cement formulation to withstand drilling and screw insertion without being excessively damaged for an appropriate time frame of >10 min after mixing.

However, no drillable bone cement is currently available on the market and there are further downsides associated with the clinically used bone cements.

Polymethylmethacrylate (PMMA) is the most commonly used polymeric bone cement as it provides a high compressive strength of over 100 MPa [4], but its limitations are an exothermal setting reaction of up to 120 °C in vitro and its lack of drillability [5]. Furthermore, PMMA is not resorbed in vivo and has an uncertain long-term stability. It is subject to cracking and migration of the stiff cement body with decreasing bone density. Consequently, PMMA is not suitable for young trauma patients. 

Viable alternatives of PMMA, especially for younger patients, include calcium phosphate cements (CPCs). CPCs do not develop the excessive heat during setting but still do not provide drillability and are uncertainly resorbed, at best within years. For most of the current commercial formulations, its mechanical strength is also too low to be applied for load bearing defects without an additional osteosynthesis [6]. In conclusion, the ideal bone cement has not been found yet.

Recently, magnesium phosphate cements (MPCs) have received broader attention as they lack the aforementioned drawbacks. MPCs provide early and high strength up to 100 MPa [3,7,8,9] and cure at <60 °C [8,9]. In contrast to PMMA and CPCs, they are presumably absorbed within months in the human organism, as can be assumed from animal studies [10] and cell studies [11]. Osteoblastic cell studies could further demonstrate a desired biocompatibility of this bone cement type [12]. 

The characteristics of MPCs were validated in previous studies demonstrating promising strength values, feasible clinical application and desired interdigitation with bone spongiosa [3,9,11,12,13]. Accordingly, MPCs could solve the challenges of low strength values, strong exothermic setting reaction and missing absorbability.

The experimental MPC formulation of our previous study [3] still missed the favorable drillability. Regarding this, one recently described approach seems encouraging. In the study of Hurle et al., 2018 [14] phytic acid (inositol hexaphosphate, IP 6, C_6_H_18_O_24_P_6_) has been shown to be an appropriate setting retardant for CPC formulations. Phytic acid delayed the viscosity increase, led to a cohesive cement paste and thus appears favorable in regard to drillability.

Consequently, we designed this study to explore if and how the addition of IP 6 can lead to a high-strength MPC with drillable characteristics. The mentioned in-house MPC from our previous study [3] is modified with various IP 6 amounts and magnesium oxide (MgO) to obtain a cohesive cement formulation allowing drilling in an appropriate time frame after injection. Via a systematic approach, IP 6 content, the amount ratio of IP 6/MgO and powder-to-liquid ratio (PLR) are varied, and the resulting formulations evaluated by an experienced trauma surgeon for clinical demands. To allow a basic characterization of the bone cements and ensure proper clinical handling, Gilmore needle test and injectability tests are performed. Further mechanical characterization includes compressive strength testing and tensile load testing by screw pullout tests. The MPCs are then applied for defect filling in an evaluated fracture model for tibial head depression fractures [2,3,15] with and without an additional osteosynthesis. The experimental set-up in terms of fracture model, laboratory and team is kept identical to our previous study [3] to allow a comparison of the various bone cements evaluated.

Therefore, the aim of this study was to develop and evaluate a new drillable, stable under biomechanical loading and probably absorbable MPC addressing the actual existing downsides of bone substitutes for clinical application.

## 2. Materials and Methods

### 2.1. Cement Fabrication

Cement powder consisted of farringtonite (Mg_3_(PO_4_)_2_) and magnesium oxide (MgO) whereas the liquid phase was formed of an aqueous solution of phytic acid (IP 6). 

To synthesize farringtonite, a mixture of 2 mol MgHPO_4_ × 3 H_2_O (newberyite) and 1 mol Mg(OH)_2_ (both Sigma Aldrich, Steinheim, Germany) was sintered for 5 h at 1050 °C in a sintering furnace (Oyten Thermotechnic, Oyten, Germany). After crushing the sintering cake, it was sieved <355 µm and dry milled for 80 min in a planetary ball mill (PM400, Retsch, Haan, Germany). Magnesium oxide and 50 wt% phytic acid solution was purchased from Magnesia GmbH (Magnesia GmbH, Lüneburg, Germany) and Sigma Aldrich, respectively. In a first step, farringtonite was manually mixed with magnesium oxide on a glass slap with a spatula. Then, IP 6 was added and after a homogenous mixture was achieved, the cement was transferred into the silicone molds or into a 10 mL syringe with a 3.5 mm outlet to be injected into the defects.

### 2.2. Pretesting Series

In a pretesting series, 13 different cement formulations were evaluated and two were found to be appropriate for clinical application.

In a first series, the IP 6 content was varied from 20.0 wt% to 25.0 wt% with a corresponding MgO content from 6.0 wt% to 7.5 wt% and the PLR was kept constant at 2.0 g/mL (Table 1, cements 1 to 3). As the setting time was too fast for clinical demands, the PLR was decreased to 1.71 g/mL in the next series (Table 1, cements 4 to 6). Finally, in the last series, the amount ratio between MgO and IP 6 was varied between 5.91 and 9.88 (Table 1, cements 7 to 13). All cements were evaluated by the same experienced orthopedic trauma surgeon in terms of handling, viscosity, injectability, setting time and interdigitation into bone spongiosa. If one of the formulations met the aforementioned criteria from a clinical point of view, compressive strength testing and screw pullout testing followed.

As cement MPC_22.5 and cement MPC_25 appeared to best comply with the clinical demands, they were chosen for further biomechanical evaluation (Table 1). 

### 2.3. X-ray Diffraction Analysis

The qualitative and quantitative phase composition of the cement raw powder and of both formulations (MPC_22.5 and MPC_25) after 3 h of setting was determined via X-ray diffraction analysis (XRD). Cuboid samples (12 mm × 6 mm × 6 mm) of set cements were ground with a pestle and mortar and an X-ray diffractometer D5005 (Siemens, Karlsruhe, Germany) with a Cu-K_∝_ radiation, a 40 kV voltage and 40 mA current were used. A 2 theta range from 20° to 40°, a step size of 0.01° and a scan rate of 1.5 s/step were applied. For the qualitative analysis, the measurement curves were compared with Joint Committee on Powder Diffraction Standards (JCPDS) reference curves. Further quantitative analysis was done by Rietveld refinement analysis with the software Topas 2.0 (Siemens, Karlsruhe, Germany). 

### 2.4. Determination of pH Profile during Setting

Cement pastes of MPC_25 and MPC_22.5 were mixed and immediately transferred into cylindrical silicon molds (15 mm diameter × 20 mm height). The pH electrode (WTW SenTix^®^ Sur, Xylem Analytics, Weilheim, Germany) was inserted into the cylindrical sample and pH value recording was done by a pH meter (inolab pH/Ion Level 2, WTW, Xylem Analytics, Weilheim Germany). Values were recorded every minute for the first 90 min and then every hour until 24 h were reached.

### 2.5. Gilmore Needle Test

**Figure 1:**
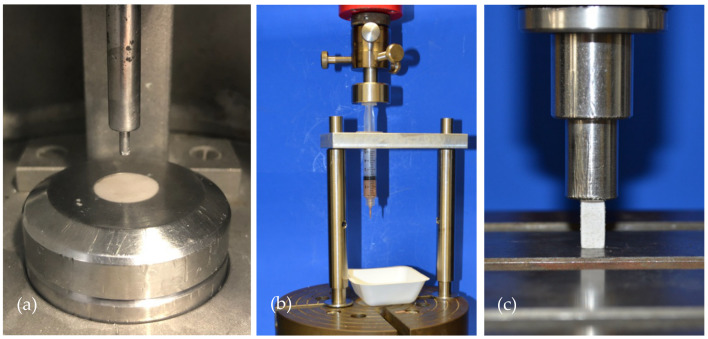
Overview of the test set-up for the Gilmore Needle Test, injectability testing and compressive strength testing. (**a**) The Gilmore Needle Test was conducted according to ASTM Standard C266-99 for both cement formulations in a custom-made humidity chamber. (**b**) Injectability testing was done by applying the load of the materials testing machine on the handle of the cement-filled syringe. (**c**) Compressive strength was done by placing cuboid samples of 12 mm × 6 mm × 6 mm under the indenter of the materials testing machine. Samples were axially loaded until failure with 1 mm/min.

To characterize the cements in terms of setting time, American Society for Testing and Materials (ASTM) Standard C266-99 Gilmore Needle Test [16] was conducted in a custom-made humidity chamber at 37 °C and >90% humidity with a needle of 113.98 g and 2.117 mm diameter (Figure 1a).

### 2.6. Injectability and Cohesion Testing

Injectability was tested by mixing and transferring the cements into a commercially available 5 mL syringe with a 2 mm outlet (Becton Dickinson, Franklin Lakes, NJ, USA), which was mounted on a custom-made apparatus in the materials testing machine (Zwick 1440, Zwick/Roell, Ulm, Germany) (Figure 1b). By conducting a load to failure test on the handle of the syringe at 20 mm/min with a peak force limited to 300 N, injectability was calculated accordingly to the following equation: $\frac{m\left(initial\right)-m\left(test\right)}{m\left(initial\right)-m\left(syringe\right)}$. Here, *m(syringe)* describes the weight of the unfilled syringe, *m(initial)* the weight of the fully filled syringe and *m(test)* the weight of the syringe after the test. To determine the cohesion of the cement pastes, the two cement formulations were injected immediately after mixing into deionized water.

### 2.7. Compressive Strength Testing

Mixed cement pastes were filled into silicon molds to obtain cuboid samples with dimensions of 12 mm × 6 mm × 6 mm. After 15 min of setting in the molds, the samples were transferred to a 37 °C water bath to allow curing for 24 h. Before testing, edges of the samples were polished using a fine sandpaper to remove burrs and the cross-sectional area was measured. Samples were then placed under the indenter of the materials testing machine (Zwick Z020, Zwick/Roell, Ulm, Germany) and the axial load to failure on the longitudinal axis of the cuboids was carried out with 1 mm/min (Figure 1c). Compressive strength was calculated as the failure load through cross-sectional area of the samples.

### 2.8. Screw Pullout Testing

Of every cement formulation, one group with embedded and one with manually drilled screws was tested in screw pullout testing (Table 2). For both groups, cortical screws (25 mm × 3.5 mm, DePuy Synthes, Umkirch, Germany) were used. 

**Figure 2:**
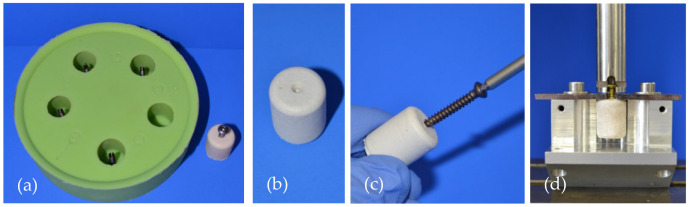
Specimen preparation and test set-up for the screw pullout tests. (**a**) Silicon mold with screws at the bottom for the preparation of embedded screw specimens. (**b**) Specimen for the groups with drilled screws. The central notch served as a marker for subsequent drilling and tapping. (**c**) Screw insertion into a specimen made of MPC_25 after 15 min of hardening. (**d**) Screw pullout testing in a custom-made device where the screw head was gripped by a holder attached to the crosshead of the materials testing machine.

In the embedded group, screws were placed through the bottom of the molds and then the cement was filled inside the form, providing a standardized embedding depth of 15 mm in the samples (Figure 2a).

For the manual insertion group, a central notch at the bottom of the forms served as a marker for the screw insertion (Figure 2b). After 15 min of pre-setting, analogous to the clinical practice, drilling (2.5 mm) and tapping (3.5 mm) were performed before the screws were inserted 15 mm deep into the samples (Figure 2c). 

The cylindrical samples of 20 mm height and 15 mm diameter were put in a 37 °C water bath for 24 h. Then, the samples were mounted on a custom-made device and axial screw pullout tests were conducted through a holder gripping the screw head with 1 mm/min (Figure 2d). Peak force of the screw pullout was recorded with the machine’s testing software testXpert II^®^ (Zwick/Roell, Ulm, Germany).

### 2.9. Analysis of the Microstructure at the Cement Screw Interface after Drilling

To analyze the changes in microstructure at the cement screw interface caused by drilling, one screw pullout specimen with embedded and one with manually drilled screw of each formulation (prepared according to Section 2.6) was potted into one-component resin (Technovit^®^ 2021 LC Fast, Kulzer, Hanau, Germany). After light-curing under visible blue light for 10 min, the samples were sanded in an ascending series with 120, 800 and 1200 grit sandpaper until the center of the samples was reached. Analysis of the interface was then performed with a stereomicroscope (Axio Zoom V16, Carl Zeiss, Jena, Germany). 

### 2.10. Testing in a Model for Tibial Head Fractures

A previously validated model [2,3,15] of tibial head depression fractures (Working Group for Osteosynthesis Questions (AO) 41-B2, Schatzker III) on synthetic osteoporotic bones (Synbone^®^ 1110, Synbone, Malans, Switzerland) was used to biomechanically evaluate the cements in a clinical test set-up. Synbones were cut at mid-diaphysis and potted at 5° valgus in commercially available gypsum in a custom-made fixation device. 

**Figure 3:**
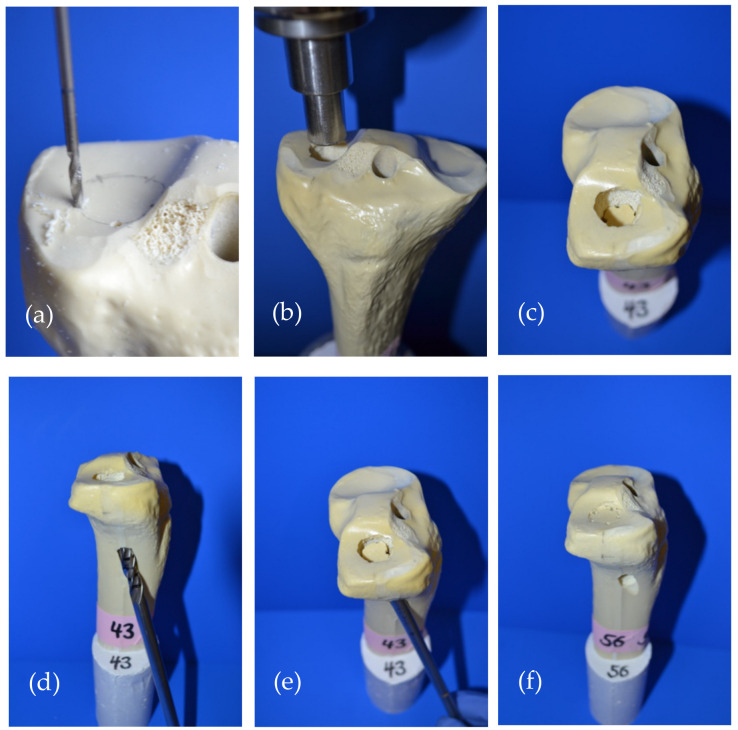
Fracture generation and subsequent fracture reduction according to the standard operation procedure for pure tibial head depression fractures is shown: (**a**) Five determined breaking points were set circularly on the lateral tibial plateau; (**b**) A pure depression fracture was generated with the 12 mm indenter of the materials testing machine; (**c**) The generated impression fracture of the lateral plateau, 12 mm in diameter and 15 mm deep; (**d**) For fracture reduction, the depressed fragment was detected with a carefully inserted k-wire, which allowed for guided drilling until the subchondral area under the fragment was reached; (**e**) After access to the subchondral area had been established, a cannulated ram was used to restore the depressed fragment to a plane articular surface; (**f**) Restored articular surface and visible drill channel which was filled with bone cement in the following steps.

After 5 determined breaking points were set circularly on the lateral plateau (Figure 3a), a 15 mm deep and 12 mm in diameter impression fracture was created by the 12 mm-indenter of the materials testing machine (Figure 3b,c). Subsequently, the fracture reduction was achieved according to the standard operation procedure for tibial depression fractures. The depressed articular fracture fragment was detected by a k-wire and a cannulated 8 mm drill (Depuy Synthes, Umkirch, Germany) was used to gain access to the subchondral area under the fragment (Figure 3d). A cannulated ram was then used to elevate the depressed fracture fragment (Figure 3e) and restore the plane articular surface of the lateral plateau (Figure 3f).

**Figure 4:**
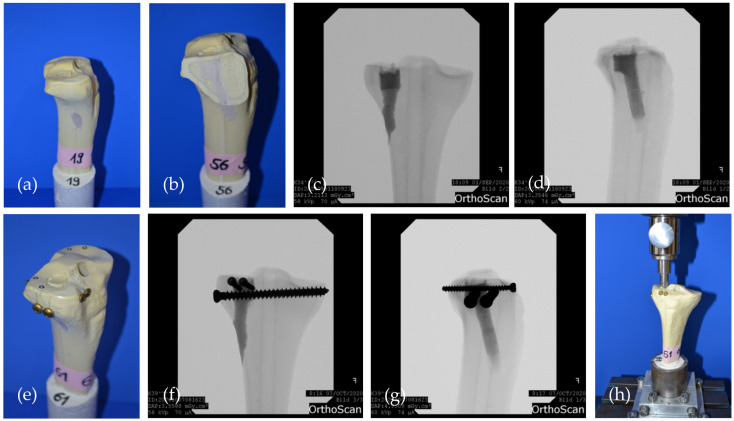
The two groups evaluated in the fracture model are shown: (**a**) Specimen where the drill channel was filled with bone cement MPC_25 and no additional screws were added; (**b**) Sagittally cut through the drill channel filled with bone cement; (**c**) Corresponding a.p. and (**d**) lateral x-rays of a specimen of the bone cement only group; (**e**) Specimen where four screws in the jail technique were drilled through the cement body after pre-setting of 15 min; (**f**) Corresponding a.p.; and (**g**) lateral x-rays of a specimen of the jail group. The screws support the reduced fracture like a grid; (**h**) Biomechanical test set-up where the load on the reduced fracture fragment is applied by the 12 mm indenter previously used for fracture generation.

In the first two groups (Table 2), the remaining bone defect of the reduction channel was then filled with the bone cement only (Figure 4a–d). In the other two groups, the bone defect was first filled up and the cement allowed to set for 15 min. Afterwards, a four screw osteosynthesis in the jail technique [17] was added: After 2.5 mm predrilling, two 3.5 mm cortical bone screws were sagittally inserted through the cement directly under the reduced fragment. Two 6.5 mm cancellous bone screws (4.5 mm predrilling) were then inserted from lateral through the cement directly under the afore set screws in order to support the reduced fracture fragment like a grid (Figure 4e–g). Anterior–posterior and lateral x-rays of the tibial head ensured accurate screw placement, and complete and equal filling of the defect.

Synbones^®^ were then kept moist with saline soaked gauzes and stored in an incubator at 37 °C for 24 h, before they were mounted again on the fixation device in the materials testing machine (Figure 4h). 

The cyclic testing phase began with 10 settling cycles of 20 to 125 N which was followed by 3000 measuring cycles with loading from 20 to 250 N. Seamlessly after the last cycle, load to failure tests were conducted with 1 mm/min. Similar to fracture generation, the load was applied by the same 12 mm-indenter of the materials testing machine on the reduced articular fracture fragment throughout the two testing phases [2,3,15,18] (Figure 4h). Displacement (mm) and maximum load (N) were recorded by the traverse of the material testing machine. 

Stiffness of the constructs was determined by calculating the ascending slope of the elastic deformation in the load–displacement curve at the beginning of the load-to-failure test of the between 150 and 500 N load. Additionally, optical markers were placed on the dorsal side of the specimen and on the fixation device to track the displacement of the lateral plateau and ensure rigid fixation of the specimens with an optical 3D metrology system (Aramis, GOM, Braunschweig, Germany). 

### 2.11. Statistical Analysis

In a previous study, impression fractures of human cadaveric tibiae were reduced with screws and with screws plus Norian^®^ bone substitute [15]. Cohen’s *d* obtained from the means and standard deviations of those two groups was 1.48, leading to a group size of nine specimens per group with β set at 0.2 and α at the conventional value of 0.05. This group size calculation was performed within a statistical expert opinion by the Institute for Mathematics and Statistics of the University of Würzburg. Furthermore, post hoc power analysis of our study from 2019 preceding this biomechanical evaluation [3] yielded a power of 99% for the displacement during the measuring cycles of the seven groups according to G*Power 3.1 statistics software (G*Power 3.1.9.6, 2020, Heinrich Heine University Düsseldorf, Düsseldorf, Germany). Consequently, the sample size of nine specimens per group was adopted for this study.

Normal distribution was checked with the Shapiro–Wilk test and by analyzing quantile-quantile plots (Q-Q-Plots). 

If normal distribution was confirmed, homogeneity of variances was checked by Levene- and Welch-Test and if so, one-way ANOVA was conducted with post hoc Tukey test. If homogeneity of variances was not obtained, the Dunnett-T3 post hoc test was performed. In case of not normally distributed data, a Kruskal–Wallis test with a subsequent Mann–Whitney U-Test was used to compare the parameters between groups. Statistical software used was SPPS Statistics^®^ 23 (SPSS Statistics 23.0.0.0, 2018, IBM, Armonk, NY, USA).

## 3. Results

### 3.1. X-ray Diffraction Analysis

**Figure 5:**
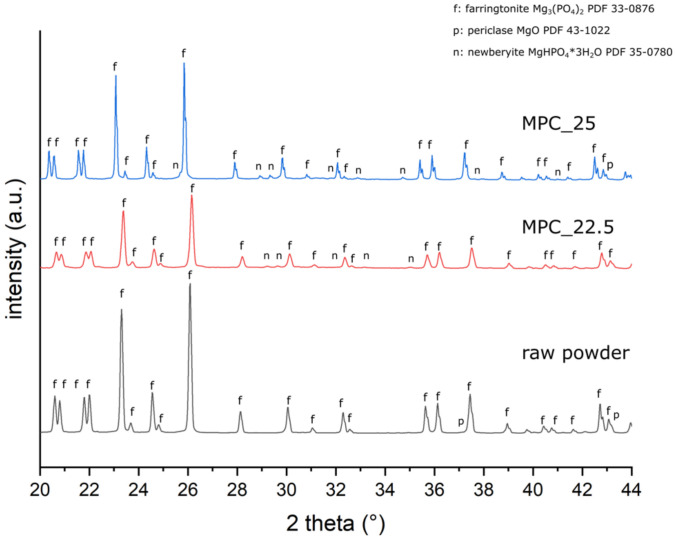
XRD patterns of the cement raw powder and of grind powder of MPC_25 and MPC_22.5 after 3 h of setting. Distinctive peaks are marked with f = farringtonite; n = newberyite; and p = periclase.

Cement raw powder (synthesized according to Section 2.1) consisted of farringtonite (Mg_3_(PO_4_)_2_) and small amounts of periclase (MgO). In both cement formulations, small amounts of periclase and newberyite (MgHPO_4_ × 3 H_2_O) were found besides farringtonite (Figure 5, Table 3).

### 3.2. Determination of pH Profile during Setting

**Figure 6:**
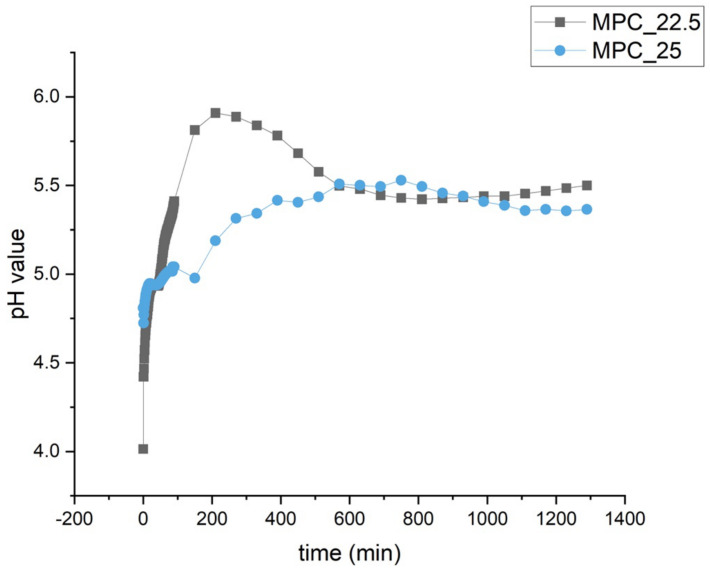
pH profile during the setting of MPC_25 and MPC_22.5. During the first 10 h, values are slightly lower for MPC_25. Both formulations approach a value of approximately 5.5 in the second half of the recording.

The pH value curve of MPC_25 showed slightly lower values during the initial 10 h than MPC_22.5. Both curves then approach a value of approximately 5.5 during the following hours (Figure 6).

### 3.3. Setting Time, Injectability and Cohesion

**Figure 7:**
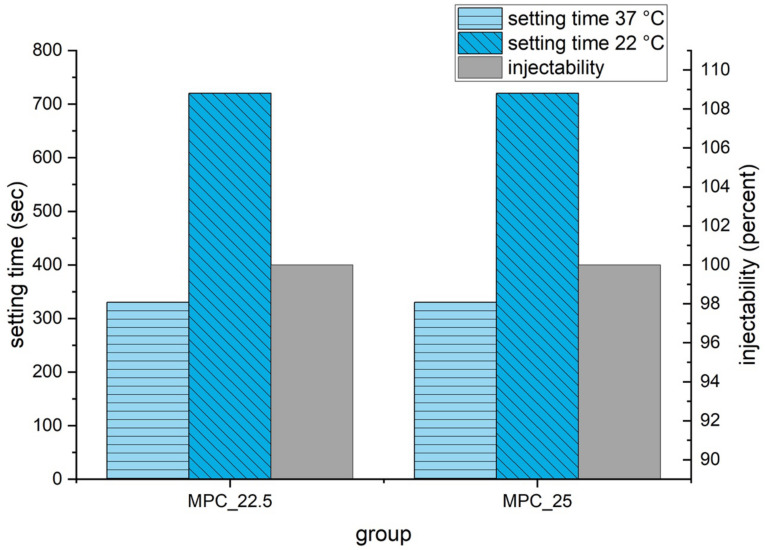
Setting time at 37 °C according to Gilmore Needle Test and at 22 °C laboratory room temperature. Injectability at 22 °C room temperature was measured with the force of the materials testing machine on the handle of the syringe limited to 300 N and is displayed in percent on the right ordinate.

**Figure 8:**
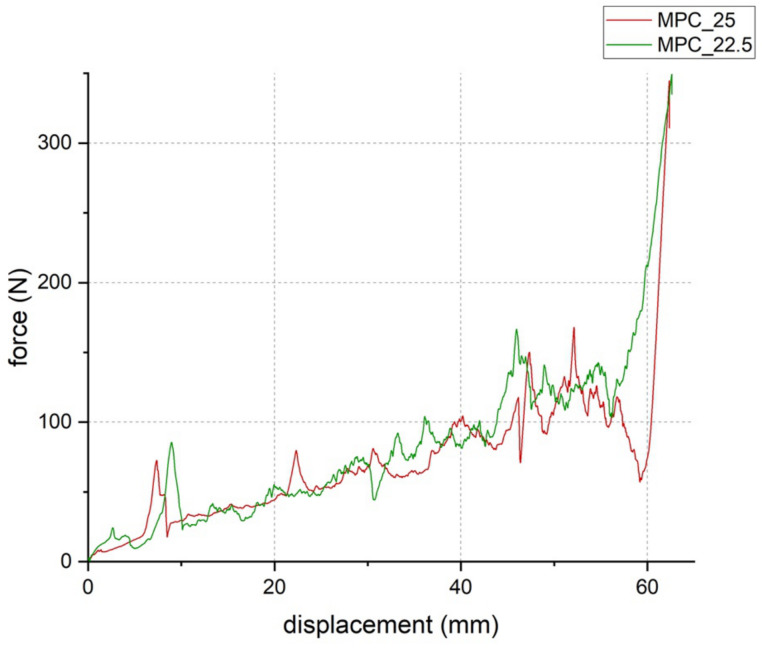
Load-to-displacement curve of MPC_22.5 (green) and MPC_25 (red) throughout the injectability test where force was applied through the traverse of the materials testing machine on the handle of the cement-filled syringe. The peak at the end of the test occurred when the cement was fully pressed out and the handle hit the outlet of the empty syringe (test set-up illustrated in Figure 1b).

**Figure 9:**
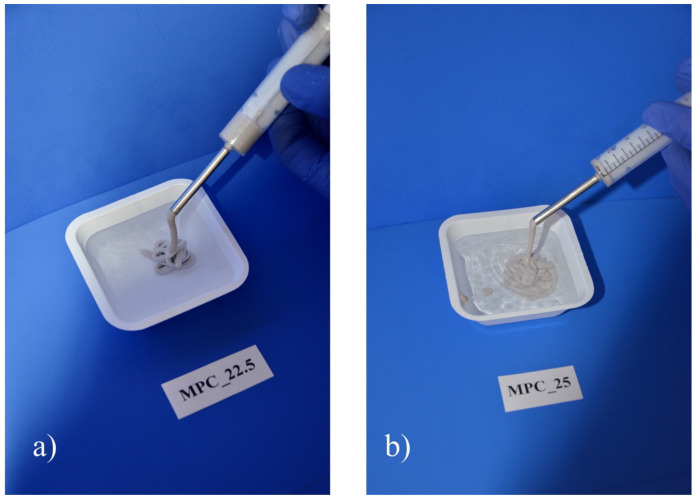
Cohesion test of the two MPC formulations. Full cohesion was achieved for both formulations, as neither (**a**) MPC_22.5 nor (**b**) MPC_25 showed streaks at the water surface when injected into deionized water.

Setting time according to the Gilmore Needle Test was 5 min 30 s for both cement formulations. At a laboratory room temperature of 22 °C, manually molding of the cement samples was possible for 12 min. During the injectability tests, both cement formulations could be injected to 100% at 22 °C (Figure 7) and showed similar load to displacement curves with forces below the maximum value of 300 N (Figure 8). MPC_25 and MPC_22.5 showed full cohesion when injected into water without any streaks on the water surface (Figure 9).

### 3.4. Compressive Strength

**Figure 10:**
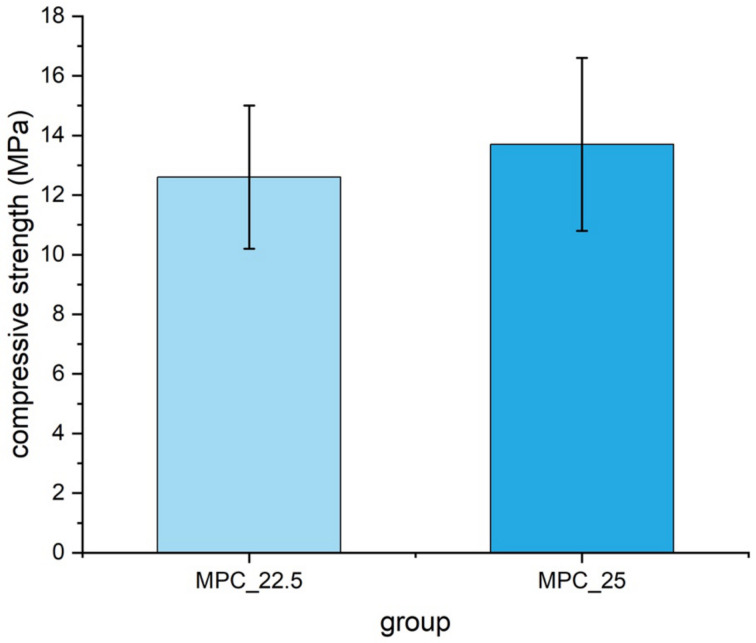
Compressive strength of cuboid samples (12 mm × 6 mm × 6 mm) made of MPC_22.5 and MPC_25, with values slightly higher for MPC_25. Differences were not statistically significant (*p* = 0.18).

Mean compressive strength was 13.7 MPa ± 2.9 MPa for cement MPC_25 and 12.6 MPa ± 2.4 MPa for cement MPC_22.5 without being statistically significant (*p* = 0.18) (Figure 10).

### 3.5. Screw Pullout Tests

**Figure 11:**
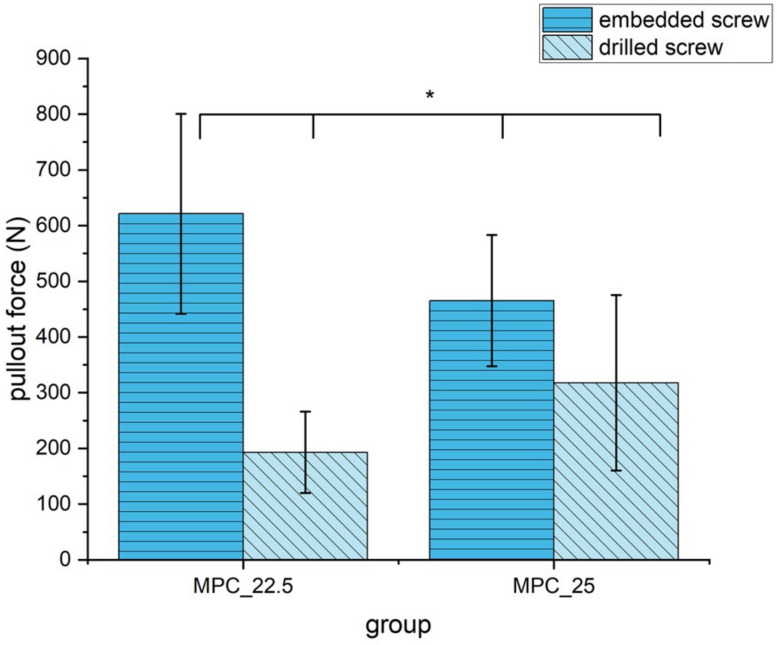
Pullout strength of the two MPC formulations with embedded and drilled screws. All differences were statistically significant with *p* < 0.05 (marked with an asterisk *).

Embedding the screws, MPC_22.5 showed a significantly higher pullout strength with 621.3 N ± 179.5 N than the other formulation MPC_25 with 465.3 N ± 117.7 N (*p* = 0.02). In contrast, if manually inserting the screws, MPC_22.5 showed a significantly lower pullout strength with 193.0 N ± 73.0 N than MPC_25 with 317.7 N ± 157.5 N (*p* = 0.04). Comparing embedded and drilled screws for MPC_22.5, pullout force was significantly reduced from 621.3 N ± 179.5 N to 193.0 N ± 73.0 N (*p* < 0.01). For MPC_25 pullout force was significantly reduced from 465.3 N ± 117.7 N to 317.7 N ± 157.5 N (*p* = 0.04). The decrease in pullout force was 69% for MPC_22.5 and 32% for MPC_25, when screws were manually inserted instead of embedded (Figure 11).

### 3.6. Analysis of the Microstructure at the Cement Screw Interface after Drilling

**Figure 12:**
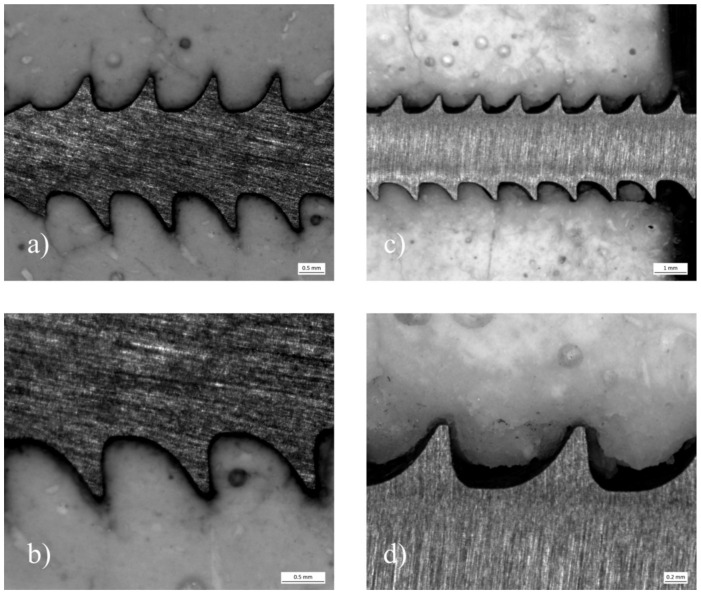
Stereomicroscope images of pullout specimens made of MPC_25 with embedded and drilled screws: (**a**) Depicts a specimen, where the screw was embedded. The cement mantle seams to interdigitate seamlessly into the screw threads. At a close-up view (**b**) only a small gap can be distinguished contrary to (**c**,**d**), where a screw was manually inserted after drilling and tapping.

Analyzing the cement screw interface between the samples with embedded and drilled screws, a visible gap could be distinguished between the screw and the surrounding cement for the specimens with drilled screws. In contrast, a seamless interdigitation of the cement mantle into the screw threads seemed to be the case for the embedded specimens (Figure 12).

### 3.7. Biomechanical Tests in a Clinically Relevant Fracture Model

#### 3.7.1. Displacement

**Figure 13:**
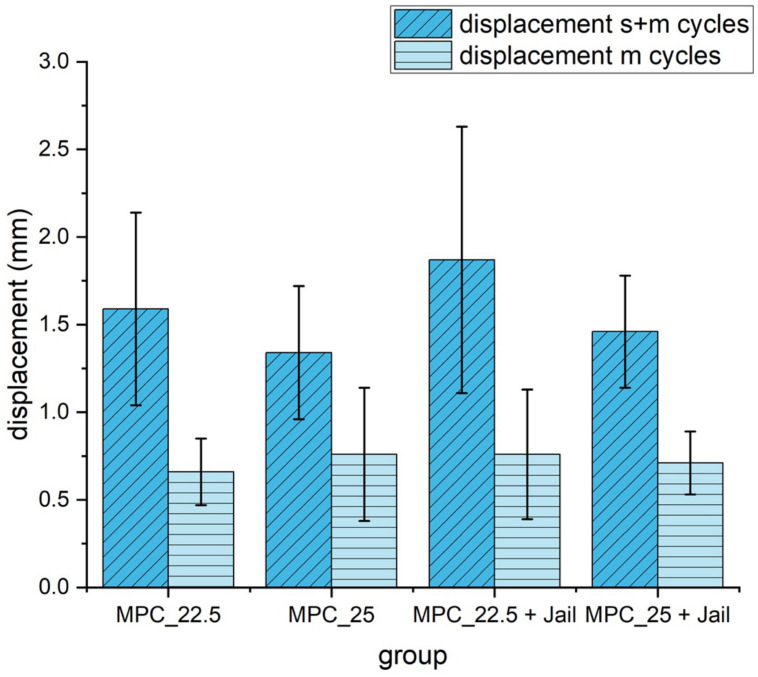
Displacement of the reduced and fixed fractures with MPC and MPC plus screws in the jail technique under cyclic loading of 10 settling cycles (20–125 N) and 3000 measuring cycles (20–250 N). Differences between displayed groups were not statistically significant (*p* = 0.17). s+m cycles = settling and measuring cycles; m = measuring cycles only.

Displacement of the reduced fracture fragment after 10 settling cycles and 3000 measuring cycles did not differ statistically significantly (*p* = 0.17). Displacement during the 3000 measuring cycles was only alike in all groups and also did not differ significantly (*p* = 0.97) (Figure 13). 

#### 3.7.2. Maximum Load

**Figure 14:**
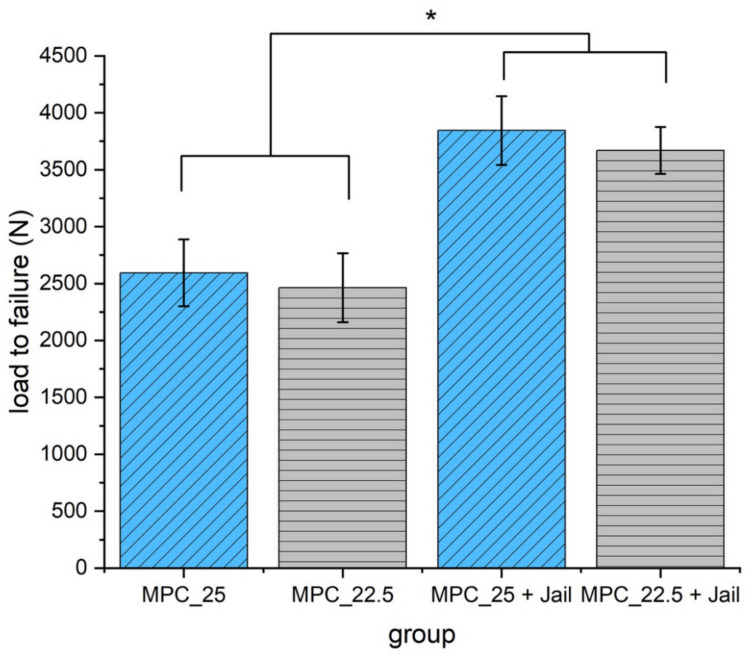
Load to failure of the reduced and fixed fractures with MPC and MPC plus screws in the jail technique. Groups with screws showed significantly higher load to failure values when compared to the groups without screws (*p* < 0.05, marked with an asterisk *). No significant difference was found within the groups with a bone substitute, only (MPC_25 vs. MPC_22.5, *p* = 0.387), and within the groups with screws (MPC_25 + Jail vs. MPC_22.5 + Jail, *p* = 0.297).

Maximum load was significantly higher in the groups with an additional four screws in the jail technique compared to the groups without osteosynthesis (*p* < 0.05) (Figure 14).

#### 3.7.3. Stiffness

**Figure 15:**
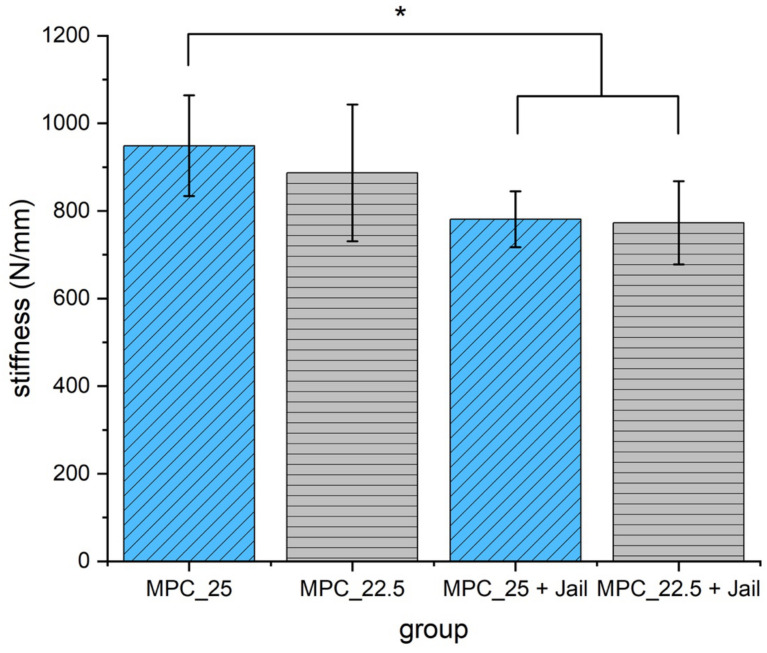
Stiffness of the reduced and fixed fractures with MPC and MPC plus screws in the jail technique. Stiffness of MPC_25 was significantly higher when compared to the two groups with additional screws (*p* < 0.05, marked with an asterisk *).

Analyzing the stiffness revealed significant higher values for MPC_25 without osteosynthesis compared to the two groups with screws (*p* < 0.05) (Figure 15). 

### 3.8. Analysis of the Optical 3D Metrology System

**Figure 16:**
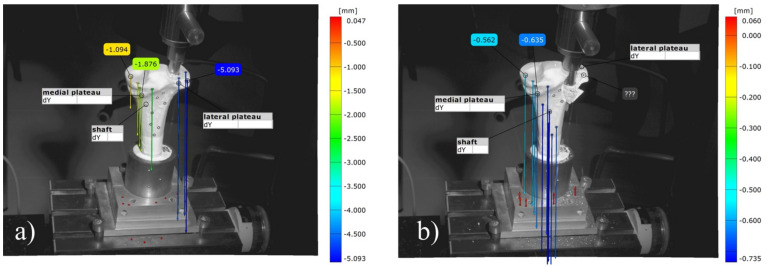
Images of the 3D metrology system of bone no. 28, bone defect filled with MPC_22.5. The most laterally placed optical reference point is also shown (blue): (**a**) Under the increasing load of the indenter, the cement is pressed out of the drill channel; (**b**) Representative failure mode for the group with bone cement only where fracture occurred on the lateral plateau with an intact tibial diaphysis.

**Figure 17:**
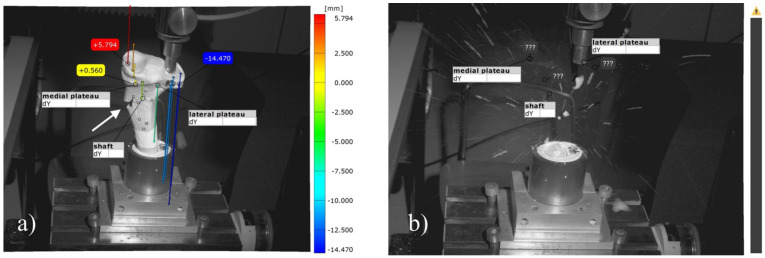
Images of the 3D metrology system of bone no. 40, the bone defect was filled with MPC_22.5 and afterwards four screws in the jail technique were added. The most laterally placed optical reference point is also shown (blue). (**a**) Contrary to the fracture pattern seen in groups without osteosynthesis, a fracture of the medial corticalis at the metaphyseal area occurred during the load to failure (white arrow in **a**); (**b**) This was followed by a fracture of the shaft whereas the tibial condyles remained intact. The image was taken at the moment of fracture, when bone debris was flying through the laboratory.

The optical tracking of the reference markers showed that the most laterally placed marker on the lateral tibial plateau revealed a higher displacement for the groups with an osteosynthesis than in those with the bone substitute only in the load-to-failure tests (Figure 16 and Figure 17). The displacement of this reference point under increasing load until failure was for MPC_25 4.46 mm ± 1.38 mm, for MPC_22.5 4.52 mm ± 0.91 mm, for MPC_22.5 + Jail 14.03 mm ± 7.35 mm and for MPC_25 + Jail 12.46 ± 3.76 mm. 

Moreover, different modes of failure could be recorded. In the groups with bone cement only, the cement body was pressed out of the drill channel until the indenter hit the lateral corticalis and a fracture of the lateral tibial plateau occurred (Figure 16). In the groups with additional screws, however, the corticalis cracked on the metaphysis under the medial plateau, and as a consequence, the entire tibial head broke away (Figure 17).

## 4. Discussion

This study presents the development of a magnesium phosphate cement (MPC) with clinically desired characteristics like drillability. It demonstrates that formulations with 22.5 wt% and 25 wt% IP 6 meet the clinical demand on drillable cement in terms of setting time, injectability, compressive strength and screw pullout strength. A typical clinical setting of a tibial head depression fracture with a metaphyseal bone defect requiring filling was selected. It could be demonstrated that the drillable IP 6 modified cements represent an alternative to other bone cements as they provide biomechanically stable fracture reduction and enable screw insertion through the cement body. Comparing the two IP 6 modified MPCs with experimental and commercial bone cements, the new cements might be clinically superior as they provide a unique combination of drillability, higher strength values and presumed absorbability.

Two different parameters evolved as crucial for the development of a clinically usable cement formulation. 

The PLR had to be reduced from 2.0 g/mL to 1.71 g/mL to provide injectability, moldability, and an appropriate setting time. It is well known that decreasing the PLR leads to an increased setting time and lower compressive strength. Additionally, the ratio between MgO as a setting accelerator and IP 6 as a setting retardant was found to be best at about 8.5 to obtain the desired drillability. When the amount ratio exceeded this value, MgO counteracted the ductile effects of IP 6. However, drilling was also not possible when the amount ratio was lower than that as the setting retardation of IP 6 led to a liquid and slowly setting cement. Consequently, MPC_22.5 and MPC_25 were chosen as they best met the clinical criteria in the aforementioned parameters and in compressive and pullout strength testing.

The XRD results supported the assumption that an amorphous end product was formed. In addition to farringtonite originating from the raw powder, small extents of periclase and newberyite were found in the XRD analysis. The quantitative amount of newberyite was higher for MPC_25, which may be explained by the higher IP 6 content, promoting more phosphate ions for newberyite formation. Periclase presumably originates from the raw powder and from the periclase added during cement mixing. As no further crystalline phase could be detected, it is likely that an amorphous end product was formed through the chelation of Mg^2+^ with phosphate groups of IP 6.

Similar to this study, it was shown in the literature that a higher IP 6 content leads to a lower pH value during setting. It could be demonstrated that increasing amounts of IP 6 in a CPC led to lower pH curves during setting the higher the IP 6 content in the cement was [14]. Therefore, the initial lower pH curve of MPC_25 is attributed to the higher IP 6 content.

In this study, a setting time of 5 min 30 s for MPC_22.5 and MPC_25 was measured, which appears too fast from a clinical point of view. However, in a laboratory room temperature of 22 °C, the cements were manually moldable for up to 12 min. Surgery occurs under air-conditioning at a temperature of 22 °C, whereas the bone defects were additionally irrigated with saline before cement injection. This means that throughout the clinical application of cements, the ambient temperature and contact surface temperature are below 37 °C with an increased and thus slower setting time. 

It has previously been shown by Hurle et al., 2018 [14] that IP 6 increases the zeta potential and thus improves the injectability of the cement paste through particle repulsion. An injectability of >93% for a CPC modified with 20 wt% IP 6 was reported [14]. This may explain the injectability of 100% in this study for the two cement formulations under a force limit of 300 N simulating the moderate manual pressure.

The cohesion test demonstrated that both formulations were cohesive enough to be injected into bony defects without being washed away by body fluid. This is a necessary characteristic for an application of the cement formulations in orthopedic trauma surgery.

The drillability of the two formulations achieved in the current study is well in agreement with the effects of IP 6 on cement systems described in other studies.

Hurle et al. [14] also showed that IP 6 leads to a chelation with divalent cations as Ca^2+^ and consequently slows down hydration speed. This leads to a slower temperature development, a delayed increase in viscosity, a ductile fracture behavior, improved injectability and increased setting time. Brueckner et al., 2019 [13] demonstrated that the effects of IP 6 not only work for CPC systems but for MPC systems as well, leading to a sticky cement paste through chelation with Mg^2+^. Consequently, IP 6 led to the desired drillable characteristics in the current study.

Regarding the compressive strength measured in this study, the increased values for MPC_25 in contrast to MPC_22.5 might be attributed to the increased IP 6 content. This could have led to quantitatively more chelation with Mg^2+^ and thus more cohesion of the cement.

Comparing the evaluated MPCs of this study with our previous study, Brueckner et al., 2019 [3], the formulations with IP 6 showed lower compressive strengths than the MPC without IP 6, from which they were derived. This might be caused by the reduced PLR, the setting retardation and formation of an amorphous end-product instead of struvite due to IP 6 [9,13]. Still, the compressive strength for the MPCs with IP 6 is higher than for the experimental drillable CPC (~6 MPa) and in the range of clinically used Graftys^®^ Quickset (~19 MPa) (Graftys, Aix en Provence, France) from our previous study. This demonstrates why the IP 6-modified MPCs are a promising solution to the downsides of clinically used bone cements. They may combine strength values of today’s used bone cements with drillability and presumed absorbability, which neither Graftys^®^ nor the drillable CPC show.

A weakening of the cements through drilling and tapping in the case of the screw pullout tests was observed. This could be further confirmed by the analysis of the pullout specimens under the stereomicroscope, where an obvious damage at the cement screw interface became visible caused by manually inserted screws. However, the pullout force loss was lower for MPC_25 compared to MPC_22.5. This can be explained by the higher IP 6 content, leading to more chelation with Mg^2+^ and consequently more cohesion and less brittleness. Thus, a slightly higher IP 6 concentration in the cement system seems beneficial.

Researching the displacement in the fracture bone model, the drillable MPCs evaluated in this study showed no significantly higher displacement when combined with screws. This is in contrast to the higher displacement which is seen when a non-drillable bone substitute is combined with screws in tibial head depression fractures [2,15]. In our previous study [3], in the group Jail + Graftys^®^, the osteosynthesis had to be performed prior to filling up the bone defect with bone cement, as Graftys^®^ is a non-drillable bone substitute. This resulted in a higher displacement compared to the group Graftys^®^ without screws, where Graftys^®^ was used as filler alone for fracture reduction. Thus, MPC_25 and MPC_22.5 demonstrated the beneficial effects of a drillable bone substitute by allowing an osteosynthesis after defect filling, which leads to a better and complete filling of the subchondral area and avoids hindering the filling process by afore placed screws.

The displacement of the reduced fracture is of particular interest as it is a crucial parameter for healing after tibial head fractures. The risk for developing posttraumatic osteoarthritis is directly linked to the ratio between the remaining articular step and the cartilage thickness [19]. The cartilage of the knee joint is known to be between 2 and 6 mm [20,21]. Brown et al., 1988 showed, that an articular cartilage step exceeding 1.5 mm after a tibial plateau fracture led to higher unphysiological stress on the cartilage as under physiological conditions [22]. Furthermore, it could be demonstrated by Honkonen et al., 1994 [23] that articular gaps exceeding 3 mm led to a deterioration in the functional outcome of patients with tibial plateau fractures.

Both MPC formulations with and without screws provided a displacement below 2 mm not exceeding the thickness of the cartilage. The displacement values of MPC_25 and MPC_25 + Jail even remained under the threshold of 1.5 mm, whereas MPC_22.5 and its combination with screws slightly exceeded this value. As a consequence, MPC_25 provided a lower displacement than other bone cements evaluated in previous studies [3] and therefore might lead to a better patient outcome after tibial head fractures.

The maximum load is also important for patient outcome in cases of postoperative high loading like full weight-bearing or falls during mobilization. Comparing the maximum load of the reduced fractures filled with MPC_22.5 and MPC_25 in this study with our analogous previous study [3], the values for both MPCs with IP 6 are higher than for any other commercial or experimental cement. This might be due to a stable interdigitation into the spongiosa of the artificial bones caused by an appropriate cement viscosity and once again due to a strong chelation between IP 6 and Mg^2+^. 

Those presumptions are further supported by the stiffness values of the MPCs, which were higher in comparison to the bone cements of our previous study, suggesting a stiff and rigid fracture fixation through the cement. In conclusion, also in the aspect of the maximum load and stiffness of the reduced fractures, the MPCs with IP 6 seem to be a feasible alternative to conventional bone cements. 

Another important conclusion for the treatment of pure tibial depression fractures can be derived from the results of the load-to-failure tests. As the maximum load at failure was higher in the groups with screws, it should be recognized that for a biomechanically stable fracture reduction, bone cement has to be combined with screws. This is further supported by the data of the optical 3D metrology system, implying that in the groups with osteosynthesis, the axial load force is transmitted from the lateral plateau to the metaphysis through the screws. The recommendation of combining screws with bone cement for the investigated fractures is a validated concept [2,18,24].

With regard to the addition of phytic acid to a cement system, there might be concerns about the biocompatibility of this setting retardant. In the study of Meininger et al., 2017 [25] various setting retardants were evaluated for their biocompatibility on osteoblasts (hFOB 1.19) and osteoclasts (murine macrophage cell line, Ralph and Williams (RAW) 264.7). Moreover, 0.1 M phytic acid added to a dicalcium phosphate cement showed a higher cell number and higher cell activity for the osteoblasts compared to the cement without setting retardant. Further findings were that phytic acid had no detrimental effect on cell activity and the differentiation of Receptor Activator of NF-kB Ligand (RANKL) treated RAW 264.7 cells on the cement surface. As phytic acid additionally improved the compressive strength and reduced the temperature peak of the setting reaction, it can be concluded that phytic acid is a biocompatible and promising setting retardant in vitro.

The resorption of MPCs in vivo has been investigated before. Kanter et al., 2014 [26] and Kanter et al., 2018 [10] found that a struvite forming MPC was almost completely resorbed within 10 months in critical size defects in the femur and tibia of sheep. Resorption occurred regardless of whether the defect was load bearing or not. In the load bearing defects, the cement withstood the load at all time points and no cracking could be observed. A loss of nearly 90% of the initial strength of the cement–bone compound could be seen after an implantation period of 4 months. Despite this comparatively quick resorption, the defects were not filled with fibrous tissue but with newly formed bone as confirmed by µCT and histomorphometry. 

Moreover, the newly formed bone exhibited thicker trabeculae than normal bone, maybe as a result of a stimulated osteoblastic activity by released Mg^2+^-Ions. 

Histologic analysis with Tartrate-resistant acid phosphatase (TRAP) staining showed a rich osteoclastic resorption and ongoing osteoblastic bone formation at the cement surface without any signs of inflammation. 

In contrast, the control defect was filled with fibrous tissue after 10 months and the hydroxyapatite control showed no signs of resorption at the end point of the study. 

Accordingly, it could be concluded, that an MPC will likely be absorbed in the human organism.

However, influences of cement additives to the resorption kinetics remain. 

Kanter et al., 2014 [26] demonstrated practically no resorption for citric acid containing brushite cements, which is in contrast to Apelt et al., 2004 [27]. The latter observed a resorption of more than 60% for a brushite cement in a similar femoral defect model in sheep. This was attributed to the setting retardant citric acid, which is known to change cell attachment to the cement matrix and thus decelerate cement resorption [28]. 

Hence, cement additives seem to play an important role in the absorption process.

As the aforementioned studies imply, a struvite forming MPC is likely to be resorbed in the human organism without inflammation and beyond that may has beneficial effects through osteoblast stimulating Mg^2+^-particles. No adverse effect on the absorption kinetics is expected through phytic acid as it has been shown to be biocompatible and stimulate cell growth of osteoblasts.

This study has limitations. The fracture model with Synbones^®^ (Synbone AG, Zizers, Switzerland) does not represent in vivo conditions where ongoing cement absorption and remodeling take place. The used Synbones^®^ 1110 osteoporotic models are only similar to human osteoporotic bone but not identical in terms of mechanical properties and in terms of the cement surrounding body fluid. Hardware and cements may be subjected to more complex forces and influence soft tissue in vivo.

## 5. Conclusions

This study demonstrates that the modification of a magnesium phosphate cement (MPC) with phytic acid creates a clinically desired drillability. This cement composition represents a feasible alternative to current experimental and commercial bone cements in basic material tests and in biomechanical tests in a model with tibial head depression fractures.

As phytic acid modified MPC unifies drillability with high strength values and presuming biocompatibility and absorbability, it is a candidate to be further evaluated in an animal testing model.

## Figures and Tables

**Table 1:** Overview of the chemical composition of all evaluated magnesium phosphate cement (MPC) formulations. wt% = weight percent; PLR = powder to liquid ratio; n = amount of substance (mol).

	wt% IP 6	wt% MgO	wt% Mg_3_(PO_4_)_2_	PLR g/mL	Amount Ratio $\frac{n\left(MgO\right)}{n\left(IP\;6\right)}$	Comment
Cement 1	**20.0**	6.0	94.0	2	9.85	setting time too fast
Cement 2	**22.5**	6.8	93.2	2	9.92	setting time too fast
Cement 3	**25.0**	7.5	92.5	2	9.85	setting time too fast
Cement 4/MPC_25	25.0	7.5	92.5	**1.71**	8.44	handling and drilling appropriate
Cement 5/MPC_22.5	22.5	6.8	93.2	**1.71**	8.50	handling and drilling appropriate
Cement 6	22.5	6.8	93.2	**1.82**	9.02	drilling impossible
Cement 7	20.0	**6.8**	93.2	1.71	**9.56**	drilling impossible
Cement 8	22.5	**4.73**	95.27	1.71	**5.91**	setting time too slow, too liquid
Cement 9	22.5	**7.2**	92.80	1.71	**9.00**	drilling impossible
Cement 10	22.5	**7.5**	92.5	1.71	**9.38**	drilling impossible
Cement 11	22.5	**7.87**	92.13	1.71	**9.84**	drilling impossible
Cement 12	25.0	**8.05**	91.95	1.71	**9.06**	drilling impossible
Cement 13	25.0	**8.78**	91.22	1.71	**9.88**	drilling impossible

**Table 2:** Overview of the test groups and sample size n.

Test Groups	MPC_22.5	MPC_25
compressive strength	n = 10	n = 10
screw pullout tests	-	-
embedded	n = 10	n = 10
manually drilled	n = 10	n = 10
fracture model	-	-
bone cement only	n = 9	n = 9
bone cement + jail-technique	n = 9	n = 9

**Table 3:** Results of the quantitative X-ray Diffraction Analysis (XRD) of the cement raw powder and of the cement formulations after 3 h of setting.

Quantitative XRD	Farringtonite (Mg_3_(PO_4_)_2_)	Periclase (MgO)	Newberyite (MgHPO_4_ × 3H_2_O)
raw powder	98.5%	1.5%	
MPC_22.5	90.8%	3.9%	5.3%
MPC_25	90.5%	2.5%	7%

## Data Availability

The video records of the optical 3D metrology system can be obtained from the corresponding author upon reasonable request.
